# A Fight against Carbolic Acid

**Published:** 1904-11

**Authors:** 


### A Fight Against Carbolic Acid.

THE *Brooklyn Medical Journal* has been waging a warfare against the unrestricted sale of carbolic acid and has carried its campaign to a successful issue, Dr. Darlington, president New York Board of Health, having issued an ordinance requiring greater care on the part of druggists in dispensing this drug. In commenting on this the *Brooklyn Medical Journal* says: "Notwithstanding a protest from representatives of the Kings County Pharmaceutical Society, the ordinance will stand. We are unable to understand the attitude of the pharmacists toward this very necessary provision. According to a report, it is claimed that carbolic acid is so commonly used for cleansing purposes that the druggists have to sell it undiluted without a physician's order. Such an argument will receive no consideration, and Dr. Darlington will be abundantly justified when statistics shall have accumulated sufficiently to show the results of his order."

Buffalo might well follow Brooklyn's example in this respect with advantage. The sale of carbolic acid and cocaine is practically free in this city, and it is not a very difficult matter to secure morphine without a physician's prescription. It is a strange state of affairs, but one which exists, nevertheless; that it is in many cases easier to get poisons at a drug store than it is to secure liquor without a prescription. One reason for this is that the state excise department punishes erring druggists, while nothing is done when a suicide is found, with an empty carbolic bottle bearing a druggist's label by his side.

---

FRANKLIN STUART TEMPLE, who was associated with "Antonius," the "Boy Wonder," in his Buffalo career, was found guilty in the Supreme Court, on October 14, of violating the ordinance, requiring registration with the Board of Health. A fine of $100 was imposed. The costs will amount to about $100. A year ago Temple was tried in the Municipal Court, found guilty and fined $250. He appealed and secured a new trial, which resulted in a second conviction. This will stand, and marks one of the closing chapters of the career of Antonius as one of the most brazen medical fakirs who ever preyed on the ignorant, the foolish, and the superstitious of Buffalo.

When the British Medical Association met at Swansea, Wales, last year, Burroughs, Wellcome & Company issued a souvenir entitled, Antient cymric medicine, which was as nearly a complete history of medicine in Wales as could be prepared. This souvenir has now been reprinted in pamphlet form and is a most interesting addition to medical literature. The pamphlet is illustrated with reproductions of early manuscript medical works of the thirteenth century, and maps and engravings showing Druidic sacrifices and instruments and scenes.

---

An invitation was extended to the members of the Medical Society of the State of New York by the president of the New York Medical Association, Dr. William H. Thornton, to attend the meeting in New York, October 18-20, 1904, of the association and participate in the scientific discussions.

The notice of this courteous action on the part of the president of the association reached us through the secretary of the society, October 15,—too late for publication in the October issue of this journal. Nevertheless, we take pleasure in mentioning the fact and in expressing the hope that the invitation was received in season for the society members to accept it.

---

University Lectures.—A lecture was given on Friday, October 21, at 4 p. m., in the new Young Men's Christian Association building, the subject being, "Popular Government."

This was the first of the university lectures in the effort to establish an academic department of the University of Buffalo.

Following the lecture there was a general discussion of practical questions affecting the city government.

---

The health department in connection with the committee of the Charity Organisation Society has issued a circular regarding tuberculosis control, in which the aid of the medical profession is asked to prevent the spread of the disease. The circular is reprinted elsewhere.

---

The councilmen have approved the resolution of the aldermen at a recent meeting in granting Dr. Walter D. Greene, health commissioner of Buffalo, authority to appoint an extra man temporarily, for the purpose of disinfecting houses in which there have been cases of tuberculosis in the last five years. This is another step forward in combating this dire malady, now that the infectious nature of tuberculosis is so universally recognised. The importance of carefully disinfecting houses where cases of this disease have occurred cannot be overestimated.

